# Exploring the neural correlates of dream phenomenology and altered states of consciousness during sleep

**DOI:** 10.1093/nc/nix009

**Published:** 2017-05-31

**Authors:** Julian Mutz, Amir-Homayoun Javadi

**Affiliations:** 1 Department of Epidemiology and Biostatistics, School of Public Health, Faculty of Medicine, Imperial College London, London W2 1PG, UK; 2 School of Psychology, Keynes College, University of Kent, Canterbury, CT2 7NP, UK

**Keywords:** sleep and dreaming, consciousness, phenomenology, neuroscience, psychosis

## Abstract

The science of dreaming constitutes a relevant topic in modern-day neuroscientific research and provides major insights into the study of human consciousness. Linking specific, universal, and regularly occurring stages of sleep with dreaming encourages the direct and systematic investigation of a topic that has fascinated humankind for centuries. In this review, we explore to what extent individuals dream during periods of rapid eye movement and non-rapid eye movement sleep, and we introduce research on lucid dreaming. We then discuss how dreaming during different stages of sleep varies in terms of phenomenological characteristics, and to what extent individuals are conscious throughout the sleep cycle. Finally, we provide a synopsis of the previous literature on brain activity during sleep, and we aim to clarify how the neurofunctional changes observed throughout sleep may lead to changes in phenomenological aspects of dreams, and in the domain of consciousness.

## Introduction

Sleep deceives by the ingenuity of its illusions, that there are no certain indications by which we may clearly distinguish wakefulness from sleep. From death, even.

          —Philip Kerr, *A Philosophical Investigation*, 1992

Dreaming may be defined as a mental state, an altered state of consciousness, which occurs during sleep. Dreams usually involve fictive events that are organized in a story-like manner, characterized by a range of internally generated sensory, perceptual, and emotional experiences (Desseilles *et al.* 2011). The world of dreams constitutes a major aspect of human experience and has both fascinated and mystified mankind since time immemorial. Much has been speculated about the origin, meaning, and purpose of dreaming, while the private nature of dreams has made an objective analysis extremely difficult (Horikawa *et al.* 2013). Early accounts of dream interpretation suggested that dreams predict the future (Artemidorus 1975) or reflect the current state of one’s mental health (Bond 1753). The scientific investigation of dreaming only emerged during the late 19th century and primarily focused on factors that influence dream content (e.g. odours) (Hervey de Saint-Denys 1867).

Throughout the past few decades, several biological and psychological theories about the purpose of dreaming have been put forward (Lavie and Hobson 1986; Barbera 2008). Whereas earlier theories by psychoanalytic scholars suggested that dreams constitute a meaningful reflection of unconscious processes ‘whose psychic importance is equal to that of the conscious mind itself’ (Jung 1934, 139), others have argued that dreams are not inherently meaningful. According to one of the most prominent theories of the origin of dreams, the activation-synthesis hypothesis, dreaming results from rapid eye movement (REM) sleep physiology (Hobson and McCarley 1977). More recent theories suggest that dreams fulfil an adaptive function related to emotion-regulation, learning, and memory consolidation (e.g. Eiser 2005; Desseilles *et al.* 2011). Dreaming may play an important role in reactivating and further consolidating novel and individually relevant experiences that occurred during waking hours (Cipolli *et al.* 2004; Schwartz 2010). It might also constitute a biological defence mechanism, which has evolved as a capacity to repeatedly simulate threatening situations (Revonsuo 2000).

In the present review, we provide an overview of the extent to which dreaming occurs during different stages of sleep and discuss how dreams vary in terms of phenomenological characteristics and consciousness. To this end, one way of studying a mechanism— such as consciousness—is to examine it in different states of functionality. This is a necessary endeavour to gather a comprehensive understanding of the overall functionality of the mechanism or process in question. We provide a synopsis of the literature that explored the neural correlates of dreaming and highlight some methodological issues in dream research. Our aim is to clarify how the neural correlates of dreams relate to changes in phenomenological characteristics and features of consciousness throughout sleep.

## Dreaming in Different Phases of Sleep

Every night individuals undergo several cycles of REM and non-rapid eye movement (NREM) sleep (further described below) that are on average 90–100 min long. Dreaming often goes unnoticed, and people tend to underestimate how often and how much they dream (Nir and Tononi 2010). This is due to our tendency to forget dreams, also known as dream amnesia (Roth *et al.* 1988).

### REM sleep

During the early 1950s, Aserinsky and Kleitman (1953) discovered REM sleep, which is characterized by REMs, global high-frequency and low amplitude electroencephalogram (EEG) activity (similar to the waking state), as well as increased heart rate, respiratory activity, and muscle atonia (i.e. temporary muscular paralysis; Jouvet 1994). In the early days of dream research, dream physiology was equated with REM sleep physiology (Aserinsky and Kleitman 1953; Dement and Kleitman 1957; Eiser 2005) because individuals are most likely to report dreams after awaking from this phase of sleep (Maquet *et al.* 1996, 2000; Nofzinger *et al.* 1997; Maquet 2000; Fox *et al.* 2013). However, it is important to note that REM sleep and dreaming can be dissociated: lesions in the forebrain can leave REM sleep intact while dreaming ceases, whereas brain stem lesions can prevent REM sleep from occurring while individuals continue to report dreams after awakening (Solms 2000).

### NREM sleep

Even though dream research has in the past mostly focused on the study of REM sleep, awakenings from NREM sleep yielded reports of dreaming as well (Foulkes 1962; Nielsen 2000; Nir and Tononi 2010; Limosani *et al.* 2011). NREM sleep is now commonly divided into three different stages (N1, N2, and N3; Iber *et al.* 2007) [N3 sleep, also known as deep sleep or slow-wave sleep, was referred to as NREM sleep stages III and IV in earlier terminology (Foulkes 1962).] and is in several ways physiologically distinct from REM sleep. NREM sleep is characterized by a global low frequency and high amplitude EEG signal, slow and regular breathing and heart rate, as well as low blood pressure. Sleep stage N1 reports frequently contain accounts of dreaming (80–90% of the time), but these reports tend to be shorter than those following periods of REM sleep (Foulkes 1966). Reports after awakenings from NREM sleep N3 contained accounts of dreaming 50–70% of the time (Nielsen 2000); only few reports contained elements of dreaming after awakenings from N3 sleep early during the night, when large slow waves are most prevalent in the EEG signal (Stickgold *et al.* 2001). Sleep inertia (i.e. the subjective feeling of grogginess following abrupt awakening) after awakening from deep sleep (NREM sleep stage N3) makes the evaluation of reports following these stages very difficult, and it is unclear to what extent individuals are conscious during this phase (Chugh *et al.* 1996).

Dreams that were reported after awakenings from NREM sleep were frequently attributed to confounding factors such as recall of dreams from REM sleep periods or waking confabulations (i.e. the unintentional production of false, distorted, or misinterpreted memories). However, it is important to note that reports of dreams after awakenings from NREM sleep are not merely a recall of dreams that occurred during the REM sleep phase (Nielsen 2000), because (1) dreaming has been reported after awakenings from the first period of NREM sleep before the occurrence of REM sleep (Nielsen 2000; Cavallero *et al.* 1992) and (2) individuals reported dreams after awaking from short naps that consisted of NREM sleep only (Suzuki *et al.* 2004; Carr and Nielsen 2015). As such, it has been suggested that dreaming during NREM sleep relates to “covert REM” brain activation processes, which occur outside polysomnographically scored REM sleep (Nielsen 2000). In line with this view, it is important to realize that wakefulness, REM, and NREM sleep are not necessarily mutually exclusive phenomena (Mahowald and Schenck 2005); sleep is far from being homogenous in terms of mental experiences. Hence, dreaming might be described along a continuum, ranging from thought-like mentation that is typical of the early stages of NREM sleep to very vivid dreams that are more typical of REM sleep (Desseilles *et al.* 2011).

### Lucid dreaming

Lucid dreaming is a rare state of sleep in which individuals achieve awareness of their own state of consciousness. According to the most frequently used sleep scoring criteria, lucid dreaming is considered being a part of REM sleep (Rechtschaffen and Kales 1968; Iber *et al.* 2007) and typically occurs during late night REM sleep periods (Voss *et al.* 2009). However, recent preliminary evidence suggests that lucid dreaming may also occur during periods of NREM sleep (Stumbrys and Erlacher 2012). Lucid dreaming has a special status compared with non-lucid REM and NREM dreaming because it is a skill that needs to be trained and occurs only rarely in untrained individuals. Dream lucidity can be achieved through metacognitive training, developing autosuggestions, external sensory stimulation, and through frequently contemplating about one’s own state of consciousness (LaBerge 1980; Hearne 1983; Purcell *et al.* 1986; Stumbrys *et al.* 2012). Lucid dreaming itself might occur in different degrees, ranging from pre-lucid reflections (i.e. a minimal awareness that one is dreaming) to deliberately controlling the dream narrative (Hearne 1982; Tyson *et al.* 1984; Barrett 1992; Kahan and LaBerge 1994). Since dream lucidity can be trained and signalized in experimental settings by means of the eye-signalling technique (LaBerge *et al.* 1981), it constitutes a promising endeavour for dream and consciousness research.

## Phenomenological Characteristics of Different Sleep Phases

Many of the typical qualities of dreaming are similar to our waking experience. Such similarities include the full range of multimodal sensory qualities, colourful visual imagery, occasionally realistic pain perception, as well as almost identical spatial organization (i.e. the experience of a real world with the dreamer being at its centre) (Windt and Noreika 2011). However, Swiss psychiatrist and founder of Analytical Psychology Carl Jung already recognized in his famous work *On the Nature of Dreams* that dreams could be distinguished from wakefulness ‘by many “bad qualities” such as lack of logic, questionable morality, uncouth form, and apparent absurdity or nonsense’ (Jung 1945, 364). Interestingly, the phenomenological characteristics of dreams in the various phases of the sleep cycle differ in several ways.

### REM sleep

*Narrative.* Particularly rich, emotional, and perceptually vivid dream experiences have been reported after awakenings from REM sleep (e.g. Foulkes 1962; Dresler *et al.* 2015). Dreaming during REM sleep typically follows loose, fanciful, and often very bizarre narratives; relates to current concerns; reflects interests, personality, and mood; draws on long-term memory; and involves social interactions (Hall and Van de Castle 1966; Domhoff 2003; Fox *et al.* 2013; Underwood 2013; Foulkes 2014). The dreamer is often uncertain about time, space, and personal identities and typically has the subjective experience of being awake (Schwartz and Maquet 2002). Reports of dreaming tend to be most elaborate and bizarre after waking up from the last period of REM sleep (Hobson 2009).

*Sensation and perception.* Dreams share similarities with experiences during wakefulness, since the perceptual modalities that are utilized most during waking hours also dominate during dreaming (Hobson 1989). Dream experiences are typically characterized by a range of visual and auditory sensations, physical activities such as self-motion or interaction with objects in the environment, and involve written and spoken language (Desseilles *et al.* 2011). Reports of awakenings from REM sleep contain significantly more accounts of sensory experiences than do reports following NREM sleep (Carr and Nielsen 2015). Tactile percepts, odours, tastes, as well as pleasure and pain are not as commonly reported following REM sleep awakenings (Hall and Van de Castle 1966; Domhoff 2003; Hobson 2009; Foulkes 2014). Oftentimes the sensational and perceptual experiences of the dream world are unlike those which occur in the world of wakefulness. Alterations from waking life experiences include sensory distortions, misidentifications of characters and places, changes in spatio-temporal integration (e.g. the integration of time and location of an event), misbinding of objects’ features, dissociation, and transpositions (e.g. frequent and abrupt changes in the dream narrative) (Desseilles *et al.* 2011).

*Emotion.* Reports following REM sleep awakenings consistently contain more emotional content than those following NREM sleep (Wamsley *et al.* 2007). Dreamers tend to report elevated levels of joy, surprise, anger, fear, and anxiety (Foulkes *et al.* 1988; Strauch and Meier 1996; Fosse *et al.* 2001), whereas sadness, guilt, and depressed affect tend to be less common. A possible explanation for this finding might be less critical self-reflection during dreams (Hobson *et al.* 2000). Since REM dream reports frequently contain fear- and anxiety-related elements (Valli and Revonsuo 2009), it has been suggested that the realistic representation of fear in dreams and nightmares serves as a threat simulation in a harmless environment in order to prepare individuals for dangerous situations in real life (Revonsuo 2000; Valli *et al.* 2005). It has also been shown that several periods of dreaming during one night may be related to the same emotional conflict (Offenkrantz and Rechtschaffen 1963).

### NREM sleep

During the sleep-onset phase, individuals frequently experience hypnagogic hallucinations while being unaware that they have already fallen asleep (Underwood 2013). These experiences share some similarities with dreams during REM sleep in terms of dream bizarreness but are typically characterized by emotional flatness (Foulkes and Vogel 1965; Vogel *et al.* 1972). They are often static (Hobson *et al.* 2000; Hobson and Pace-Schott 2002) and usually involve no self-character (Foulkes 2014). Activities that were performed before sleeping might influence the content of such hallucinations (Stickgold *et al.* 2000; Wamsley *et al.* 2010).

After the sleep-onset, NREM dreams are typically more thought-like, fragmentary, and related to current concerns, unlike the vivid, hallucinatory, and mainly visual content of REM dreams (Eiser 2005). After awakenings from sleep stage N3 early during the night, reports tend to be short, thought-like, less vivid, less visual, less motorically animated, less emotional, and less emotionally pleasant than REM reports, while being more conceptual, more plausible, more concerned with current issues, and typically involve greater volitional control (Rechtschaffen 1973; Hobson *et al.* 2000). Late night NREM sleep reports are usually longer and more hallucinatory, often indistinguishable from REM sleep reports (Monroe *et al.* 1965; Antrobus *et al.* 1995).

### Similarities with psychosis

Interestingly, dream phenomenology has often been compared with psychosis because dreams share large similarities with many of the typical characteristics of psychosis and particularly with the positive symptoms of schizophrenia (e.g. false beliefs due to incorrect inferences about reality or distorted sensory perceptions that have no apparent external source) (Hobson 1989; Hobson *et al.* 2000; Hobson and Pace-Schott 2002; Limosani *et al.* 2011; Windt and Noreika 2011; D’Agostino *et al.* 2013). These similarities range from internally generated, vivid imagery to intensified and often inappropriate affect as well as diminished ego functions (i.e. the capacity to distinguish what is occurring in one’s mind and what is occurring in the outside world). Most pronounced are elevated levels and uncritical acceptance of cognitive bizarreness, decreased reality testing, and the delusional belief of being awake while dreaming (Limosani *et al.* 2011). Furthermore, the dreamer lacks control of dream events and often shows blunted distinction between first- and third-person perspectives (Hobson *et al.* 1998; Schwartz and Maquet 2002; Maquet *et al.* 2005). These observations are supported by functional magnetic resonance imagining data of psychotic patients, which suggest that dream bizarreness (i.e. improbability, incongruity, and vagueness) largely overlaps with patients’ incongruous and bizarre waking experience (for review, see Limosani *et al.* 2011). In fact, there are remarkable similarities in terms of cognitive bizarreness between the waking thoughts of individuals with psychosis, their dream reports, and the dream reports of healthy individuals (e.g. Scarone *et al.* 2008). Interestingly though, the psychotic patients differ from the healthy individuals in that they tend to judge their dream reports as less bizarre (Lusignan *et al.* 2009); they fail to distinguish self-generated and non-self-generated percepts and perceive cognitive bizarreness as being real without any critical reflection (D’Agostino *et al.* 2013).

Contrasting the state of lucid dreaming with psychosis, Dresler *et al.* (2015) suggested that understanding lucidity during dreams may shed light on the mechanisms underlying the lack of insight into the delusional nature of one’s current state of consciousness that patients with psychosis often suffer from. Taken together, these findings indicate that there might be a shared mechanism responsible for some of the features of dreaming and psychosis (Limosani *et al.* 2011). However, a recent paper by Mota *et al.* (2016) challenges this notion: psychotic lucid dreamers reported more frequent control of their dreams than non-psychotic lucid dreamers. As such, psychosis could potentially amplify the experience of internal stimuli at the expenses of external ones, enabling psychotic patients to better control their internal reality than healthy individuals.

## Changes in Level and Quality of Consciousness during Sleep

‘Just as the psyche has a diurnal side which we call consciousness, so also it has a nocturnal side: the unconscious psychic activity which we apprehend as dreamlike fantasy’ (Jung 1934, 147). In line with this notion, Jung (1945, 364) suggested that dreams are ‘fragment[s] of involuntary psychic activity, just conscious enough to be reproducible in the waking state.’ Even though this illustrates that dreaming and wakefulness likely differ with regards to conscious experience, advances in cognitive neuroscience and dream research reveal that these differences are not as clear-cut as originally assumed but can—in a simplified model—be placed along a continuum (more complex models assume a space of multiple dimensions; Voss and Voss 2014; Bayne *et al.* 2016). These range from no consciousness to simple awareness of perception and emotion (i.e. primary consciousness) to self-reflective awareness, abstract thinking, volition, and metacognition (i.e. secondary consciousness, also referred to as higher-order consciousness or self-consciousness) (Morin 2006). [It should be noted that a number of different models concerning the idea of levels of consciousness have been proposed: for instance, the four-level structural model of cognition by Brown (1976), Neisser’s (1997) five-level model of consciousness, a three-level model by Schooler (2002), or Block’s (1995) notion of phenomenal and access consciousness. In the present review, we use the concepts of primary and secondary consciousness to represent the general notions of sensory awareness and higher level of awareness such as reflection, respectively. These concepts fit the purpose of our distinction between different states of consciousness during sleep (Edelman 1992); they have been frequently used in the sleep and dream research literature (Edelman 2003; Hobson and Voss 2011; Kahan and LaBerge 2011; D’Agostino *et al.* 2013; Voss *et al.* 2014; Zink and Pietrowsky 2015).]

One can generally differentiate between consciousness during wakefulness, consciousness during dreaming, and non-consciousness, with the possibility of intermediate states being present (Limosani *et al.* 2011). Consciousness during waking hours is characterized by the awareness of the external world, our bodies, and our selves. When people are dreaming, they are to some extent consciously aware of their internal world, have sensory, perceptual, and emotional experiences but typically fail to recognize their own condition, the bizarre features of the dream world, their poor memory access, and their limited thought capabilities (Hobson 2009). Dream consciousness and waking consciousness may differ in terms of their origin (i.e. their respective causal pathway), with the former partly representing an offline, internally generated simulation of the latter. In line with this notion, it has been suggested that dreams may be seen as a purer form of consciousness, which is free of the constraints imposed by the perception of, and interaction with, physical environments (Revonsuo 2006).

Early reports suggested a single-mindedness and isolation during dreaming, which refers to the dreamer being absorbed in the dream world without being aware of an alternative reality (Rechtschaffen 1978). However, the nature, level, and quality of conscious experience during sleep show large variability (Nir and Tononi 2010). To some extent, this might be comparable to the multiplicity of conscious substates that occur during wakefulness (e.g. task vs. default modes of the brain) (Singer and Antrobus 1965; Raichle and Mintun 2006). Consciousness is clearly not an all-or-nothing phenomenon but a multifaceted concept with aspects varying across species, vigilance states (i.e. the degree of responsiveness to stimuli), and health conditions.

Although there is likely a consensus that consciousness exists while individuals are dreaming, there is an ongoing debate as to whether consciousness exists during dreamless sleep as well (Windt *et al.* 2016). Some authors argue that consciousness continues throughout dreamless sleep, provided that one remains aware of having slept. The topic of dreamless sleep is beyond the scope of this paper (for further discussions on this issue, please see Thompson 2015; see also Windt 2015).

### REM sleep

Dreams that occur during REM sleep show mostly aspects of primary but not of secondary consciousness. During REM sleep, the dreamer tends to have less metacognitive activity (i.e. the processes by which individuals monitor and control their own cognitive processes), reflective thought, and volitional capabilities (Kahan *et al.* 1997; Kahan and LaBerge 2011; Dresler *et al.* 2014). The dreamer has only limited access to information about the past and anticipated future, and typically concerns him or herself exclusively with the present content of the dream narrative (Fox *et al.* 2013). However, some reports of REM dreams involve reflective thought, such as puzzlement about improbable or impossible events, contemplative alternatives in decision-making, and reflection during social interactions (Wolman and Kozmová 2007), as well as theory of mind processes (i.e. the ability to attribute mental states to oneself and others) (Kahn and Hobson 2005).

### NREM sleep

Reports of conscious experience across NREM sleep phases vary to a great extent (Rechtschaffen 1973). Although the frequency of reporting dreams after NREM awakenings is sparse and generally less elaborate than after periods of REM sleep, which might be due to the brain’s inability to encode memories of the dreams (Massimini *et al.* 2005), the very existence of NREM dream reports provides evidence for the idea that consciousness does not fully cease during NREM sleep (e.g. Goodenough *et al.* 1965; Suzuki *et al.* 2004).

### Lucid dreaming

Lucid dreaming is a hybrid state of consciousness with features of both waking and dreaming (e.g. hallucinatory dream activity combined with aspects of primary and secondary consciousness such as self-reflective thought, abstract thinking, metacognition, and agentive control) (Voss *et al.* 2009). Dresler *et al.* (2014) recently investigated how volitional aspects of consciousness vary across wakefulness, non-lucid, and lucid dreaming. They found that levels of self-determination (i.e. the subjective experience of acting freely according to one’s will) were similar for lucid dreaming and wakefulness while being reduced in periods of non-lucid dreaming. Furthermore, planning ability (i.e. how well organized one pursues plans and intentions) seemed to be impaired during both non-lucid and lucid dreaming. However, this may be because it is not necessary to plan during dreams and spontaneous execution of intentions is simply more common. Intention enactment (i.e. how promptly and determined intentions are executed) was most pronounced during lucid dreaming and did not differ between wakefulness and non-lucid dreaming. This seems plausible because the lucid dreamer is aware that obstacles in dreams are not real and can easily be overcome (Dresler *et al.* 2014). In line with this, it has been suggested that restored access to metacognitive abilities and memory functions during lucid dreaming enable the dreamer to execute his or her intentions (Metzinger 2004; Windt and Metzinger 2007). Contrasting lucid dreaming and non-lucid dreaming mirrors contrasting primary and secondary consciousness (Dresler *et al.* 2009). Non-lucid REM sleep dreams lack those very features of secondary consciousness, which are the defining characteristic of dream lucidity. These include insight, control over though and actions, as well as logical thought (Voss *et al.* 2013). Interestingly, lucid dreaming may be the only phenomenon that can be utilized to examine changes in primary and secondary consciousness in the same vigilance level (Spoormaker *et al.* 2010) and therefore constitutes an important topic of research.

## Brain Activity during Sleep

Establishing a link between dreaming and its underlying neurofunctional changes constitutes a major challenge for researchers (Limosani *et al.* 2011) because dreaming arises from brain activity that is largely independent of interactions with external stimuli (Revonsuo 2006). Dream research typically aims to retrospectively correlate neural activity with the dream characteristics that are common to all dreams (e.g. dream bizarreness—although varying tremendously depending on the sleep stage) rather than the content of individual dreams (Nir and Tononi 2010; but see Siclari *et al.* 2017). Periods of REM sleep, NREM sleep, and lucid dreaming are characterized by patterns of regional brain activity that are both similar and distinct from those observed during wakefulness. In what follows, we review studies on brain activity during REM and NREM sleep rather than brain activity during REM and NREM ‘dreaming’ per se. As such, it is important to note that the interpretations of these findings are to some extent speculative, given that the methods used in the majority of these studies do not allow for a separation of the duration of REM/NREM sleep and dreaming.

### REM sleep

**Figure 1.**
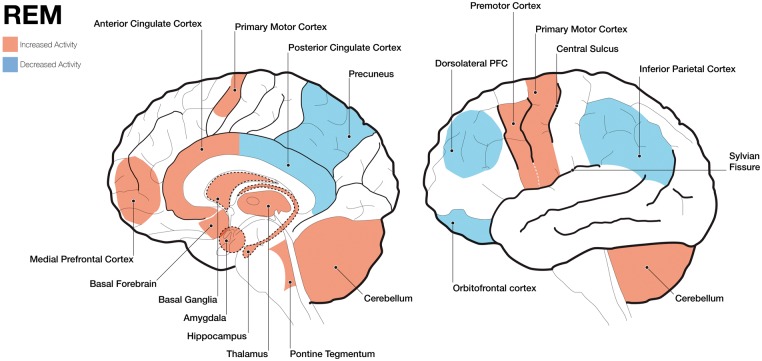
Schematic brain images showing increased and decreased activity of different brain areas during rapid eye movement (REM) sleep. The left panel shows a mid-line incision looking at the right hemisphere and the right panel shows a lateral surface of the brain. Areas highlighted with dashed borders are deeper structures.

The REM sleep phase has most clearly been defined in terms of neurofunctional activation (Fig. 1), which corresponds to some of the key characteristics of the subjective experience of dreaming (e.g. vivid imagery as well as articulate and incongruous storylines) (Limosani *et al.* 2011).

*Similarities with wakefulness.* The EEG signal during REM sleep shares large similarities with that of wakefulness, and positron emission tomography (PET) studies have shown that global brain metabolism tends to be very similar as well (Hobson *et al.* 2000; Maquet 2000).

*Hyperactivity.* Several brain regions become particularly active during REM sleep. There is strong metabolic activity in higher-order occipito-temporal visual association areas, which might be responsible for the often very vivid visual dream imagery during REM sleep (Braun *et al.* 1997; Nofzinger *et al.* 1997; Maquet *et al.* 2000). Hyperactivity in motor regions such as the primary motor and premotor cortices, the cerebellum, and the basal ganglia may account for the frequently reported motor content of dreams (Braun *et al.* 1997; Maquet *et al.* 2000). Furthermore, increased levels of activity have been observed in the pontine tegmentum, the thalamus, the basal forebrain, as well as in limbic and paralimbic structures (e.g. amygdaloid complexes, hippocampal formation, and anterior cingulate cortex) (Maquet *et al.* 1996; Braun *et al.* 1997; Nofzinger *et al.* 1997). These brain regions are associated with emotional processing and might be responsible for the often very intense emotional aspects of REM sleep dreaming (Maquet and Phillips 1998; Hobson *et al.* 2000). There is also increased activity in other regions such as the medial prefrontal cortex, circuits of the medial temporal lobe region, and the posterior cingulate cortex (Maquet *et al.* 1996; Braun *et al.* 1997, 1998; Nofzinger *et al.* 1997; Fox *et al.* 2013), which are implicated in memory and self-referential processing (Nofzinger *et al.* 1997; Ioannides *et al.* 2009). In fact, there is striking overlap between the default mode network (i.e. the network of brain regions that are active when an individual is awake and not currently engaged in a task), which is associated with self-referential processing, and areas that become increasingly active during REM sleep (Fox *et al.* 2013). This network may play a key role in both mind-wandering and dreaming and possibly represents a shared neural substrate of the two phenomena (Domhoff and Fox 2015).

*Hypoactivity.* Even though several brain regions become hyperactive during REM sleep, a number of structures show decreased levels of activity. Among these structures is the right inferior parietal cortex, which is involved in waking volition (Goldberg *et al.* 2008; Desmurget *et al.* 2009) and which contributes to a unified representation of self and self versus other perspectives (Farrer *et al.* 2003). Decreased activity of the right inferior parietal cortex (Maquet *et al.* 1996; Braun *et al.* 1997) might allow the dreamer to participate in both first- and third-person perspectives (Maquet *et al.* 2005). Moreover, there is deactivation of executive regions of the prefrontal cortex such as the dorsolateral prefrontal cortex (DLPFC) and the orbitofrontal cortex, but also in regions including the posterior cingulate gyrus, the precuneus, and the inferior parietal cortex. These areas are typically involved in cognitive control, metacognition, and ego functions (e.g. orientation in time and space, reality testing, and self-monitoring) and may underlie the lack of insight, restricted volitional capacities, and impaired metacognition during dreaming (Maquet *et al.* 1996; Braun *et al.* 1997; Nofzinger *et al.* 1997; Maquet 2000, 2005; Hobson and Pace-Schott 2002; Schwartz and Maquet 2002; Fox *et al.* 2013). Hypoactivation of the prefrontal cortex may also be a contributing factor for dream amnesia (Fox *et al.* 2013).

### NREM sleep

**Figure 2.**
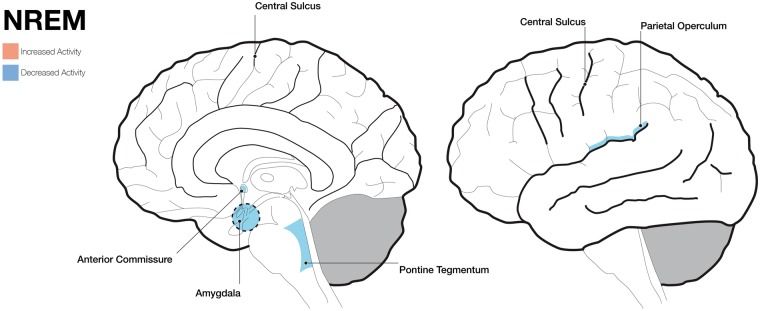
Schematic brain images showing increased and decreased activity of different brain areas during non-rapid eye movement (NREM) sleep. The left panel shows a mid-line incision looking at the right hemisphere and the right panel shows a lateral surface of the brain. Areas highlighted with dashed borders are deeper structures.

The neuroscientific study of NREM dreaming (Fig. 2) only emerged more recently, but findings tend to be more informative with regards to exploring the neural correlates of dreaming because of methodological and data-analytical advances including the use of computational learning algorithms. A recent study by Horikawa *et al.* (2013) which utilized machine learning techniques showed that visual imagery during sleep onset is represented by brain regions including the early visual pathway, fusiform face area, and parahippocampal place area. Brain activity underlying these hypnagogic hallucinations may differ from that underlying dreams occurring during REM sleep though (Underwood 2013). Utilizing high-density EEG recordings and performing serial awakenings, Siclari *et al.* (2017) showed that dream reports following awakenings from the N2 stage were preceded by ‘decreased’ low-frequency and ‘increased’ high-frequency power in bilateral parieto-occipital areas including the medial and lateral occipital lobes as well as the precuneus and posterior cingulate gyrus (for high-frequency power, the lateral frontal and temporal cortices showed increased activity as well). Furthermore, the authors confirmed these findings for sleep stages N2 and N3 in an independent sample and irrespective of the dreamer’s ability to remember specific dream contents.

### Lucid dreaming

**Figure 3.**
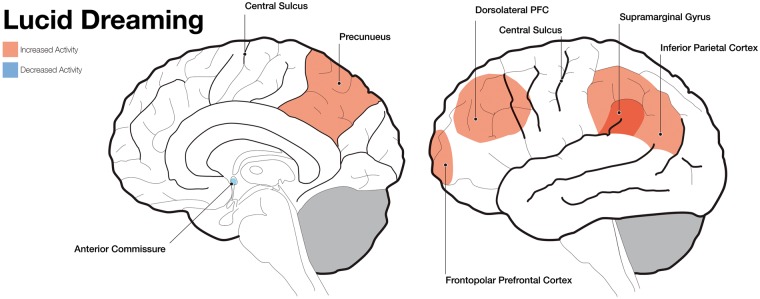
Schematic brain images showing increased and decreased activity of different brain areas during lucid dreaming. The left panel shows a mid-line incision looking at the right hemisphere and the right panel shows a lateral surface of the brain.

Periods of lucid dreaming show increased activity in areas such as the DLPFC, bilateral frontopolar prefrontal cortex, and parietal areas including the precuneus, the inferior parietal lobules, and the supramarginal gyrus (Fig. 3). It has been suggested that the fronto-parietal activity during lucid dreaming corresponds to the reinstantiation of reflective capabilities (Dresler *et al.* 2015), thereby mediating features of secondary consciousness (Dresler *et al.* 2012) such as metacognitive evaluation (Stuss *et al.* 2001; Schmitz *et al.* 2004; Fleming *et al.* 2012) and self-referential processing (Cavanna and Trimble 2006). In fact, research on the neural correlates of lucid dreaming might be key in understanding the neural substrates of secondary consciousness (Hobson and Voss 2010; Dresler *et al.* 2014). So far, the study of lucid dreaming is limited to periods of REM sleep because the classical method to investigate lucid dreaming, the eye-signalling technique, is not applicable to NREM sleep (Dresler *et al.* 2015). Furthermore, research on lucid dreaming mostly pertains to case studies, and conclusions about the neural correlates of lucid dreaming should be regarded as preliminary.

## Neural Correlates of Dream Phenomenology and Changes in Consciousness

The heterogeneous brain activity during sleep may explain some of the typical features of, and variation across, dreams during different stages of sleep (Desseilles *et al.* 2011). This is in line with Pace-Schott (2010) who described dreaming as ‘an imprecise experiential simulacrum [i.e. a representation or imitation] of waking resulting from neurobiological processes that must differ from those that generate waking consciousness.’

### Consciousness

Decreased metabolic activity in the DLPFC during periods of REM sleep might be responsible for reduced features of secondary consciousness, which return during periods of lucid dreaming when the DLPFC becomes more active again (Voss *et al.* 2009; Hobson and Voss 2010; Dresler *et al.* 2012). During REM sleep dreaming, the dreamer thinks that he or she is awake, which is a delusion that might be due to persistent inactivation of frontal and parietal circuits necessary for waking memory, self-reflective awareness, and insight. The global deactivation of the pontine tegmentum, the amygdala, the anterior commissure, the parietal operculum, deep frontal white matter, and the mid-line thalamus during NREM sleep compared with waking, and their subsequent reactivation during REM sleep might explain why some aspects of consciousness during REM sleep might be more readily available than during NREM sleep (Maquet *et al.* 1996; Braun *et al.* 1997; Nofzinger *et al.* 1997). However, preliminary findings suggest that localized activity changes in the parieto-occipital region, irrespective of global activity in the rest of the cortex, may constitute a marker of conscious experience during sleep (Siclari *et al.* 2017). The neural correlates of lucid dreaming largely overlap with brain areas that are involved in self-reflective thought and subserve volitional capabilities (Voss *et al.* 2009; Hobson and Voss 2010; Dresler *et al.* 2014).

### Phenomenology

According to the principle of perceptual equivalence (Finke 1989), there are common neural substrates for perception and imagery. Some of the visual experiences during the sleep onset phase are represented by brain activity patterns that are similar to the ones observed during stimulus perception (Horikawa *et al.* 2013), and activation in visual-occipital and auditory-temporal cortices during REM sleep may underlie the visual and auditory elements that are frequently reported after awakenings from REM sleep (Braun *et al.* 1997). In line with this, patients with extrastriate occipito-temporal lesions report cessation of visual dream imagery (Solms 1997). Several other behaviours share similar neural correlates during wakefulness and dreaming (Erlacher and Schredl 2008; Siclari *et al.* 2017). For instance, dream hand movements activate the same areas of the motor cortex that are active when actually executing hand movements during wakefulness (Dresler *et al.* 2011). Since a range of subcortical and neocortical structures that are active during waking are also active during REM sleep, but inactive during NREM sleep, this might explain why the phenomenological experience of dreaming during REM sleep is much more diverse than during NREM sleep (Hobson 2009). For instance, waking emotional processes that are active during REM sleep are inhibited during NREM sleep, and mechanisms governing sensory vividness of imagery are enhanced in REM sleep while being inhibited during periods of NREM sleep (Carr and Nielsen 2015).

## Conclusion and Future Directions

In what follows we will highlight some important shortcomings that have emerged from reviewing the current literature on the neural correlates of dreaming and we will suggest potential ways to address these in future research.

1. Given that most research has focused on the study of REM sleep, much remains to be learned about the mechanisms underlying dreaming during NREM sleep. Future investigations should more systematically contrast the different phases of NREM sleep with wakefulness, REM sleep, and periods of lucid dreaming. Recent efforts in dream research have investigated whether daytime naps are suitable for sampling and comparing dreaming during REM and NREM sleep. It was shown that recall rates for REM and NREM naps were slightly elevated to normal night-time recall rates (Carr and Nielsen 2015), making it perhaps an even more efficient tool to study dreams.
2. Causal links have—until recently—not received much attention in the literature on dreaming. Novel brain stimulation methods including transcranial alternating current stimulation (tACS) and transcranial magnetic stimulation can be used to make causal inferences on the relationship between brain activity and behaviour (e.g. Nieminen *et al.* 2016). For example, tACS has recently been utilized to experimentally induce lucid dreaming (Noreika *et al.* 2010; Stumbrys *et al.* 2013; Voss *et al.* 2014), and might hence be a valuable tool for a more systematic study of the neural substrates of dreaming.
3. No objective assessment (or verification) of dreams is currently possible and researchers rely exclusively on the collection of subjective dream reports. These are being obtained only after a change in vigilance state has occurred, which might alter the quality and content of the report (Kussé *et al.* 2010). Novel brain imaging methods, in terms of both hardware (e.g. functional near-infrared spectroscopy, fNIR or fNIRS; Dresler *et al.* 2011), and software (statistical learning or machine learning algorithms; Huth *et al.* 2016; Wen *et al.* 2016, preprint; Zurawel *et al.* 2016) may enable a more objective readout of the dream. This is particularly important considering that (i) memory is reconstructive and there is a time lag between the actual dream and its report, (ii) the original dream content may be distorted due to interfering material of the waking environment, (iii) there are limitations inherent to verbal reports, and (iv) moral censorship (Hobson *et al.* 2000; Windt 2013). Moreover, the absence of dream reports does not necessarily imply an absence of dreaming, since these are easily and quickly forgotten.
4. At present, most researchers are working in highly specialized fields (e.g. research on sleep and lucid dreaming), potentially overlooking knowledge in other relevant areas (e.g. coma and vegetative state) that could benefit their own discipline. To gain a more comprehensive understanding of the mechanisms underlying consciousness, cross-fertilization between multiple fields is necessary.
5. Some evidence suggests that dream lucidity training can be applied in clinical settings as a form of nightmare therapy (Spoormaker *et al.* 2003; Spoormaker and Van Den Bout 2006). Although the mechanism of action of lucid dreaming is not fully understood, examining the characteristics of patients for whom this type of therapy seems effective may shed light on the nature of lucid dreaming itself.
6. Understanding the mechanisms underlying consciousness in the healthy human brain might enable us to identify processes that are dysfunctional in pathologies, thereby enabling us to develop better treatments. For instance, lucid dreaming might be of therapeutic value in sleep disorders and for individuals experiencing frequent nightmares. At the same time, studies on the neural correlates of dreaming in clinical populations are informative: for example, a recent study by Dodet *et al.* (2015) has shown that comparing individuals with and without narcolepsy can elucidate the nature of lucid dreaming.

Due to limited space, only a small number of studies on pathological states were included in the present review. Moreover, research on altered states of consciousness that occur during meditation (Lutz *et al.* 2007), hypnosis (Rainville and Price 2003), vegetative states (Owen *et al.* 2006), coma (Laureys and Schiff 2012), or drug use (Carhart-Harris *et al.* 2014) could not be discussed here.

The study of dreaming is of major importance insofar as it helps to more fully understand consciousness. It offers a unique opportunity to gain insight into the processes and mechanisms involved in waking consciousness through studying the similarities with, and differences from, dream consciousness. However, we have only just started to understand the neural correlates of dreaming.

## References

[nix009-B1] Antrobus J, Kondo T, Reinsel R Dreaming in the late morning: summation of REM and diurnal cortical activation. Conscious Cogn 1995;4:275–99. doi:10.1006/ccog.1995.1039. 7497109 10.1006/ccog.1995.1039

[nix009-B2] Artemidorus D. Oneirocritica: The Interpretation of dreams (R. J. White, Trans.). Saddle River, NJ: Noyes Publications, 1975.

[nix009-B3] Aserinsky E, Kleitman N. Regularly occurring periods of eye motility, and concomitant phenomena, during sleep. Science 1953;118:273–4. doi:10.1126/science.118.3062.273. 13089671 10.1126/science.118.3062.273

[nix009-B4] Barbera J. Sleep and dreaming in Greek and Roman philosophy. Sleep Med 2008;9:906–10. doi:10.1016/j.sleep.2007.10.010. 19014776 10.1016/j.sleep.2007.10.010

[nix009-B5] Barrett D. Just how lucid are lucid dreams? Dreaming 1992;2:221–8. doi:10.1037/h0094362.

[nix009-B6] Bayne T, Hohwy J, Owen AM. Are there levels of consciousness? Trends Cogn Sci 2016;20:405–13. doi:10.1016/j.tics.2016.03.009. 27101880 10.1016/j.tics.2016.03.009

[nix009-B7] Block N. On a confusion about a function of consciousness. Behav Brain Sci 1995;18:227–47. doi:10.1017/s0140525x00038188.

[nix009-B8] Bond J. An Essay on the Incubus, or Night-Mare. London, UK: D. Wilson and T. Durham, 1753.

[nix009-B9] Braun AR, Balkin T, Wesenten N Regional cerebral blood flow throughout the sleep-wake cycle. An H2 (15) O PET study. Brain 1997;120:1173–97. doi:10.1093/brain/120.7.1173. 9236630 10.1093/brain/120.7.1173

[nix009-B10] Braun AR, Balkin TJ, Wesensten NJ Dissociated pattern of activity in visual cortices and their projections during human rapid eye movement sleep. Science 1998;279:91–5. doi:10.1126/science.279.5347.91. 9417032 10.1126/science.279.5347.91

[nix009-B11] Brown JW. Consciousness and pathology of language In: Rieber RW (ed.), The Neuropsychology of Language. London, UK: Plenum Press, 1976, 67–93. doi:10.1007/978-1-4684-2292-4_4.

[nix009-B12] Carhart-Harris RL, Leech R, Hellyer PJ The entropic brain: a theory of conscious states informed by neuroimaging research with psychedelic drugs. Front Hum Neurosci 2014;8:20. doi:10.3389/fnhum.2014.00020. 24550805 10.3389/fnhum.2014.00020 PMC3909994

[nix009-B13] Carr M, Nielsen T. Daydreams and nap dreams: content comparisons. Conscious Cogn 2015;36:196–205. doi:10.1016/j.concog.2015.06.012. 26164253 10.1016/j.concog.2015.06.012

[nix009-B14] Cavallero C, Cicogna P, Natale V Slow wave sleep dreaming. Sleep 1992;15:562–6. doi:10.1093/sleep/15.6.562. 1475572 10.1093/sleep/15.6.562

[nix009-B15] Cavanna AE, Trimble MR. The precuneus: a review of its functional anatomy and behavioural correlates. Brain 2006;129:564–83. doi:10.1093/brain/awl004. 16399806 10.1093/brain/awl004

[nix009-B16] Chugh DK, Weaver TE, Dinges DF. Neurobehavioral consequences of arousals. Sleep 1996;19:S198–201. doi:10.1093/sleep/19.suppl_10.s198. 9085510 10.1093/sleep/19.suppl_10.s198

[nix009-B17] Cipolli C, Fagioli I, Mazzetti M Incorporation of presleep stimuli into dream contents: evidence for a consolidation effect on declarative knowledge during REM sleep? J Sleep Res 2004;13:317–26. doi:10.1111/j.1365-2869.2004.00420.x. 15560766 10.1111/j.1365-2869.2004.00420.x

[nix009-B18] D’Agostino A, Castelnovo A, Scarone S. Dreaming and the neurobiology of self: recent advances and implications for psychiatry. Front Psychol 2013;4:680. doi:10.3389/fpsyg.2013.00680. 24133470 10.3389/fpsyg.2013.00680 PMC3783843

[nix009-B19] Dement W, Kleitman N. The relation of eye movements during sleep to dream activity: an objective method for the study of dreaming. J Exp Psychol 1957;53:339–46. doi:10.1037/h0048189. 13428941 10.1037/h0048189

[nix009-B20] Desmurget M, Reilly K, Richard N Movement intention after parietal cortex stimulation in humans. Science 2009;324:811–3. doi:10.1126/science.1169896. 19423830 10.1126/science.1169896

[nix009-B21] Desseilles M, Dang-Vu TT, Sterpenich V Cognitive and emotional processes during dreaming: a neuroimaging view. Conscious Cogn 2011;20:998–1008. doi:10.1016/j.concog.2010.10.005. 21075010 10.1016/j.concog.2010.10.005

[nix009-B22] Dodet P, Chavez M, Leu-Semenescu S Lucid dreaming in narcolepsy. Sleep 2015;38:487–97. doi:10.5665/sleep.4516. 25348131 10.5665/sleep.4516 PMC4335518

[nix009-B23] Domhoff GW. The Scientific Study of Dreams: Neural Networks, Cognitive Development, and Content Analysis. Washington, DC: American Psychological Association, 2003. doi:10.1037/10463-000.

[nix009-B24] Domhoff GW, Fox KC. Dreaming and the default network: a review, synthesis, and counterintuitive research proposal. Conscious Cogn 2015;33:342–53. doi:10.1016/j.concog.2015.01.019. 25723600 10.1016/j.concog.2015.01.019

[nix009-B25] Dresler M, Eibl L, Fischer CF Volitional components of consciousness vary across wakefulness, dreaming and lucid dreaming. Front Psychol 2014;4. doi:10.3389/fpsyg. 2013.00987. 10.3389/fpsyg.2013.00987 PMC3877766 24427149

[nix009-B26] Dresler M, Koch SP, Wehrle R Dreamed movement elicits activation in the sensorimotor cortex. Curr Biol 2011;21:1833–7. doi:10.1016/j.cub.2011.09.029. 22036177 10.1016/j.cub.2011.09.029

[nix009-B27] Dresler M, Wehrle R, Spoormaker V Neural correlates of consciousness—insights from sleep imaging. Neuroforum 2009;15:T24–23C.

[nix009-B28] Dresler M, Wehrle R, Spoormaker VI Neural correlates of dream lucidity obtained from contrasting lucid versus non-lucid REM sleep: a combined EEG/fMRI case study. Sleep 2012;35:1017–20. doi:10.5665/sleep.1974. 22754049 10.5665/sleep.1974 PMC3369221

[nix009-B29] Dresler M, Wehrle R, Spoormaker VI Neural correlates of insight in dreaming and psychosis. Sleep Med Rev 2015;20:92–9. doi:10.1016/j.smrv.2014.06.004. 25092021 10.1016/j.smrv.2014.06.004

[nix009-B30] Edelman GM. Bright Air, Brilliant Fire: On the Matter of the Mind. New York, NY: Basic Books, 1992.

[nix009-B31] Edelman GM. Naturalizing consciousness: a theoretical framework. Proc Natl Acad Sci U S A 2003;100:5520–4. doi:10.1073/pnas.0931349100. 12702758 10.1073/pnas.0931349100 PMC154377

[nix009-B32] Eiser AS. Physiology and psychology of dreams. Semin Neurol 2005;25:97–105. doi:10.1055/s-2005-867078. 15798942 10.1055/s-2005-867078

[nix009-B33] Erlacher D, Schredl M. Do REM (lucid) dreamed and executed actions share the same neural substrate? Int J Dream Res 2008;1:7–14. doi:10.11588/heidok.00008421.

[nix009-B34] Farrer C, Franck N, Georgieff N Modulating the experience of agency: a positron emission tomography study. Neuroimage 2003;18:324–33. doi:10.1016/s1053-8119(02)00041-1. 12595186 10.1016/s1053-8119(02)00041-1

[nix009-B35] Finke RA. Principles of Mental Imagery. Cambridge, MA: The MIT Press, 1989.

[nix009-B36] Fleming SM, Dolan RJ, Frith CD. Metacognition: computation, biology and function. Phil Trans R Soc Lond B Biol Sci 2012;367:1280–6. doi:10.1098/rstb.2012.0021. 22492746 10.1098/rstb.2012.0021 PMC3318771

[nix009-B37] Fosse R, Stickgold R, Hobson JA. The mind in REM sleep: reports of emotional experience. Sleep 2001;24:947–55. doi:10.1093/sleep/24.8.1. 11766165

[nix009-B38] Foulkes D. Dream reports from different stages of sleep. J Abnorm Soc Psychol 1962;65:14–25. doi:10.1037/h0040431. 13894288 10.1037/h0040431

[nix009-B39] Foulkes D. The Psychology of Sleep. New York, NY: Scribner, 1966.

[nix009-B40] Foulkes D. Dreaming: A Cognitive-Psychological Analysis. Abingdon-on-Thames, UK: Routledge, 2014.

[nix009-B41] Foulkes D, Sullivan B, Kerr NH Appropriateness of dream feelings to dreamed situations. Cogn Emot 1988;2:29–39. doi:10.1080/02699938808415227.

[nix009-B42] Foulkes D, Vogel G. Mental activity at sleep onset. J Abnorm Psychol 1965;70:231–43. doi:10.1037/h0022217. 14341704 10.1037/h0022217

[nix009-B43] Fox KC, Nijeboer S, Solomonova E Dreaming as mind wandering: evidence from functional neuroimaging and first-person content reports. Front Human Neurosci 2013;7. doi:10.3389/fnhum.2013.00412 10.3389/fnhum.2013.00412 PMC3726865 23908622

[nix009-B44] Goldberg I, Ullman S, Malach R. Neuronal correlates of “free will” are associated with regional specialization in the human intrinsic/default network. Conscious Cogn 2008;17:587–601. doi:10.1016/j.concog.2007.10.003. 18082425 10.1016/j.concog.2007.10.003

[nix009-B45] Goodenough DR, Lewis HB, Shapiro A Dream reporting following abrupt and gradual awakenings from different types of sleep. J Pers Soc Psychol 1965;2:170–9. doi:10.1037/h0022424. 14316977 10.1037/h0022424

[nix009-B46] Hall CS, Van de Castle RL. The content analysis of dreams In Berelson B. (ed.), The Analysis of Communication Content. New York, NY: Appleton-Century-Crofts, 1966, 147–158.

[nix009-B47] Hearne KM. Effects of performing certain set tasks in the lucid-dream state. Percept Mot Skills 1982;54:259–62. doi:10.2466/pms.1982.54.1.259.

[nix009-B48] Hearne KM. Lucid dream induction. J Mental Imagery 1983;7:19–23.

[nix009-B49] Hervey de Saint-Denys M-J-L. *Les Rêves et les moyens de les diriger*, 1867.

[nix009-B50] Hobson JA. The Dreaming Brain. New York, NY: Basic Books, 1989.

[nix009-B51] Hobson JA. REM sleep and dreaming: towards a theory of protoconsciousness. Nat Rev Neurosci 2009;10:803–13. doi:10.1038/nrn2716. 19794431 10.1038/nrn2716

[nix009-B52] Hobson JA, McCarley RW. The brain as a dream state generator: an activation-synthesis hypothesis of the dream process. Am J Psychiatry 1977;134:1335–48. doi:10.1176/ajp.134.12.1335. 21570 10.1176/ajp.134.12.1335

[nix009-B53] Hobson JA, Pace-Schott EF. The cognitive neuroscience of sleep: neuronal systems, consciousness and learning. Nat Rev Neurosci 2002;3:679–93. doi:10.1038/nrn915. 12209117 10.1038/nrn915

[nix009-B54] Hobson JA, Pace-Schott EF, Stickgold R. Dreaming and the brain: toward a cognitive neuroscience of conscious states. Behav Brain Sci 2000;23:793–842. doi:10.1017/s0140525x00003976. 11515143 10.1017/s0140525x00003976

[nix009-B55] Hobson JA, Stickgold R, Pace-Schott EF. The neuropsychology of REM sleep dreaming. Neuroreport 1998;9:R1–R14. doi:10.1097/00001756-199802160-00033. 9512371 10.1097/00001756-199802160-00033

[nix009-B56] Hobson A, Voss U. Lucid dreaming and the bimodality of consciousness In: Perry EK (ed.), New Horizons in the Neuroscience of Consciousness. Amsterdam, NL: John Benjamins Publishing Company, 2010, 155–166. doi:10.1075/aicr.79.21hob.

[nix009-B57] Hobson JA, Voss U. A mind to go out of: reflections on primary and secondary consciousness. Conscious Cogn 2011;20:993–7. doi:10.1016/j.concog.2010.09.018. 20965750 10.1016/j.concog.2010.09.018

[nix009-B58] Horikawa T, Tamaki M, Miyawaki Y Neural decoding of visual imagery during sleep. Science 2013;340:639–42. doi:10.1126/science.1234330. 23558170 10.1126/science.1234330

[nix009-B59] Huth AG, Lee T, Nishimoto S Decoding the semantic content of natural movies from human brain activity. Front Syst Neurosci 2016;10:81. doi:10.3389/fnsys.2016.00081. 27781035 10.3389/fnsys.2016.00081 PMC5057448

[nix009-B60] Iber C, Ancoli-Israel S, Chesson A The AASM Manual for the Scoring of Sleep and Associated Events: Rules, Terminology and Technical Specifications. Westchester, IL: American Academy of Sleep Medicine, 2007.

[nix009-B61] Ioannides AA, Kostopoulos GK, Liu L MEG identifies dorsal medial brain activations during sleep. Neuroimage 2009;44:455–68. doi:10.1016/j.neuroimage.2008.09.030. 18950718 10.1016/j.neuroimage.2008.09.030

[nix009-B62] Jouvet M. Paradoxical sleep mechanisms. Sleep 1994;17:S77–83. doi:10.1093/sleep/17.suppl_8.s77. 7701205 10.1093/sleep/17.suppl_8.s77

[nix009-B63] Jung CG. The practical use of dream-analysis. Collected Works 1934;16:139–62. doi:10.1515/9781400851003.139.

[nix009-B64] Jung CG. On the nature of dreams. Collected Works 1945;8:363–79. doi:10.1515/9781400850952.281.

[nix009-B65] Kahan TL, LaBerge S. Lucid dreaming as metacognition: implications for cognitive science. Conscious Cogn 1994;3:246–64. doi:10.1006/ccog.1994.1014.

[nix009-B66] Kahan TL, LaBerge S, Levitan L Similarities and differences between dreaming and waking cognition: an exploratory study. Conscious Cogn 1997;6:132–47. doi:10.1006/ccog.1996.0274. 9170565 10.1006/ccog.1996.0274

[nix009-B67] Kahan TL, LaBerge SP. Dreaming and waking: similarities and differences revisited. Conscious Cogn 2011;20:494–514. doi:10.1016/j.concog.2010.09.002. 20933437 10.1016/j.concog.2010.09.002

[nix009-B68] Kahn D, Hobson A. Theory of mind in dreaming: awareness of feelings and thoughts of others in dreams. Dreaming 2005;15:48–57. doi:10.1037/1053-0797.15.1.48.

[nix009-B69] Kussé C, Muto V, Mascetti L Neuroimaging of dreaming: state of the art and limitations. Int Rev Neurobiol 2010;92:87–99. doi:10.1016/s0074-7742(10)92005-9. 20870064 10.1016/S0074-7742(10)92005-9

[nix009-B70] LaBerge SP. Lucid dreaming as a learnable skill: a case study. Percept Mot Skills 1980;51:1039–42. doi:10.2466/pms.1980.51.3f.1039.

[nix009-B71] LaBerge SP, Nagel LE, Dement WC Lucid dreaming verified by volitional communication during REM sleep. Percept Mot Skills 1981;52:727–32. doi:10.2466/pms.1981.52.3.727. 24171230 10.2466/pms.1981.52.3.727

[nix009-B72] Laureys S, Schiff ND. Coma and consciousness: paradigms (re) framed by neuroimaging. Neuroimage 2012;61:478–91. doi:10.1016/j.neuroimage.2011.12.041. 22227888 10.1016/j.neuroimage.2011.12.041

[nix009-B73] Lavie P, Hobson JA. Origin of dreams: anticipation of modern theories in the philosophy and physiology of the eighteenth and nineteenth centuries. Psychol Bull 1986;100:229–40. doi:10.1037/0033-2909.100.2.229. 3532158

[nix009-B74] Limosani I, D’agostino A, Manzone ML The dreaming brain/mind, consciousness and psychosis. Conscious Cogn 2011;20:987–92. doi:10.1016/j.concog.2010.11.014. 21288741 10.1016/j.concog.2010.11.014

[nix009-B75] Lusignan F-A, Zadra A, Dubuc M-J Dream content in chronically-treated persons with schizophrenia. Schizophrenia Res 2009;112:164–73. doi:10.1016/j.schres.2009.03.032. 10.1016/j.schres.2009.03.032 19409757

[nix009-B76] Lutz A, Dunne JD, Davidson RJ. Meditation and the neuroscience of consciousness In Zelazo PD, Moscovitch M, Thompson E (eds.), The Cambridge Handbook of Consciousness. Cambridge, UK: Cambridge University Press, 2007, 499–555. doi:10.1017/cbo9780511816789.020.

[nix009-B77] Mahowald MW, Schenck CH. Insights from studying human sleep disorders. Nature 2005;437:1279–85. doi:10.1038/nature04287. 16251953 10.1038/nature04287

[nix009-B78] Maquet P. Functional neuroimaging of normal human sleep by positron emission tomography. J Sleep Res 2000;9:207–32. doi:10.1046/j.1365-2869.2000.00214.x. 11012860 10.1046/j.1365-2869.2000.00214.x

[nix009-B79] Maquet P. Current status of brain imaging in sleep medicine. Sleep Med Rev 2005;9:155–6. doi:10.1016/j.smrv.2005.01.003. 15893246 10.1016/j.smrv.2005.01.003

[nix009-B80] Maquet P, Laureys S, Peigneux P Experience-dependent changes in cerebral activation during human REM sleep. Nat Neurosci 2000;3:831–6. doi:10.1038/77744. 10903578 10.1038/77744

[nix009-B81] Maquet P, Peters J, Aerts J Functional neuroanatomy of human rapid-eye-movement sleep and dreaming. Nature 1996;383:163–6. doi:10.1038/383163a0. 8774879 10.1038/383163a0

[nix009-B82] Maquet P, Phillips C. Functional brain imaging of human sleep. J Sleep Res 1998;7:42–7. doi:10.1046/j.1365-2869.7.s1.7.x. 9682193 10.1046/j.1365-2869.7.s1.7.x

[nix009-B83] Maquet P, Ruby P, Maudoux A Human cognition during REM sleep and the activity profile within frontal and parietal cortices: a reappraisal of functional neuroimaging data. Prog Brain Res 2005;150:219–595. doi:10.1016/s0079-6123(05)50016-5. 16186026 10.1016/S0079-6123(05)50016-5

[nix009-B84] Massimini M, Ferrarelli F, Huber R Breakdown of cortical effective connectivity during sleep. Science 2005;309:2228–32. doi:10.1126/science.1117256. 16195466 10.1126/science.1117256

[nix009-B85] Metzinger T. Being no One: The Self-Model Theory of Subjectivity: Cambridge, MA: The MIT Press, 2004.

[nix009-B86] Monroe LJ, Rechtschaffen A, Foulkes D Discriminability of REM and NREM reports. J Pers Soc Psychol 1965;2:456–60. doi:10.1037/h0022218. 14333325 10.1037/h0022218

[nix009-B87] Morin A. Levels of consciousness and self-awareness: a comparison and integration of various neurocognitive views. Conscious Cogn 2006;15:358–71. doi:10.1016/j.concog.2005.09.006. 16260154 10.1016/j.concog.2005.09.006

[nix009-B88] Mota NB, Resende A, Mota-Rolim SA Psychosis and the control of lucid dreaming. Front Psychol 2016;7. doi:10.3389/fpsyg.2016.00294. 10.3389/fpsyg.2016.00294 PMC4783408 27014118

[nix009-B89] Neisser U. The roots of self-knowledge: perceiving self, it, and thoua. Ann N Y Acad Sci 1997;818:19–33. doi:10.1111/j.1749-6632.1997.tb48243.x. 10.1111/j.1749-6632.1997.tb48243.x 9237463

[nix009-B90] Nielsen TA. A review of mentation in REM and NREM sleep: “covert” REM sleep as a possible reconciliation of two opposing models. Behav Brain Sci 2000;23:851–66. doi:10.1017/s0140525x0000399x. 11515145 10.1017/s0140525x0000399x

[nix009-B91] Nieminen JO, Gosseries O, Massimini M Consciousness and cortical responsiveness: a within-state study during non-rapid eye movement sleep. Sci Rep 2016;6:30932. doi:10.1038/srep30932. 27491799 10.1038/srep30932 PMC4974655

[nix009-B92] Nir Y, Tononi G. Dreaming and the brain: from phenomenology to neurophysiology. Trends Cogn Sci 2010;14:88–100. doi:10.1016/j.tics.2009.12.001. 20079677 10.1016/j.tics.2009.12.001 PMC2814941

[nix009-B93] Nofzinger EA, Mintun MA, Wiseman M Forebrain activation in REM sleep: an FDG PET study. Brain Res 1997;770:192–201. doi:10.1016/s0006-8993(97)00807-x. 9372219 10.1016/s0006-8993(97)00807-x

[nix009-B94] Noreika V, Windt JM, Lenggenhager B New perspectives for the study of lucid dreaming: from brain stimulation to philosophical theories of self-consciousness. Int J Dream Res 2010;3:36–45. doi:10.11588/ijodr.2010.1.586.

[nix009-B95] Offenkrantz W, Rechtschaffen A. Clinical studies of sequential dreams: I. A patient in psychotherapy. Arch Gen Psychiatry 1963;8:497–508. doi:10.1001/archpsyc.1963.01720110073009. 13939529 10.1001/archpsyc.1963.01720110073009

[nix009-B96] Owen AM, Coleman MR, Boly M Detecting awareness in the vegetative state. Science 2006;313:1402. doi:10.1126/science.1130197. 16959998 10.1126/science.1130197

[nix009-B97] Pace-Schott EF. The neurobiology of dreaming In Kryger MH, Roth T, Dement WC (eds.), Principles and Practice of Sleep Medicine. Philadelphia, PA: Saunders, 2010. doi:10.1016/b0-72-160797-7/50051-3.

[nix009-B98] Purcell S, Mullington J, Moffitt A Dream self-reflectiveness as a learned cognitive skill. Sleep 1986;9:423–437. doi:10.1093/sleep/9.3.423. 3764289 10.1093/sleep/9.3.423

[nix009-B99] Raichle ME, Mintun MA. Brain work and brain imaging. Annu Rev Neurosci 2006;29:449–476. doi:10.1146/annurev. neuro. 29.051605. 112819. 16776593 10.1146/annurev.neuro.29.051605.112819

[nix009-B100] Rainville P, Price DD. Hypnosis phenomenology and the neurobiology of consciousness. Int J Clin Exp Hypn 2003;51:105–29. doi:10.1076/iceh.51.2.105.14613. 12908747 10.1076/iceh.51.2.105.14613

[nix009-B101] Rechtschaffen A. Psychophysiology of mental activity during sleep In McGuigan F (ed.), The Psychophysiology of Thinking. Cambridge, MA: Academic Press, 1973, 153–205. doi:10.1016/b978-0-12-484050-8.50011-x.

[nix009-B102] Rechtschaffen A. The single-mindedness and isolation of dreams. Sleep 1978;1:97–109. doi:10.1093/sleep/1.1.97. 227026 10.1093/sleep/1.1.97

[nix009-B103] Rechtschaffen A, Kales A. A Manual of Standardized Terminology, Techniques and Scoring System for Sleep Stages of Human Subjects. Washington, DC: Public Health Service, US Government Printing Office, 1968.

[nix009-B105] Revonsuo A. The reinterpretation of dreams: an evolutionary hypothesis of the function of dreaming. Behav Brain Sci 2000;23:877–901. doi:10.1017/s0140525x00004015. 11515147 10.1017/s0140525x00004015

[nix009-B106] Revonsuo A. Inner Presence: Consciousness as a Biological Phenomenon. Cambridge, MA: The MIT Press, 2006.

[nix009-B107] Roth T, Roehrs T, Zwyghuizen-Doorenbos A Sleep and memory In: Hindmarch I, Ott H (eds.), Benzodiazepine Receptor Ligands, Memory and Information Processing. New York, NY: Springer, 1988, 140–5. doi:10.1007/978-3-642-73288-1_10.

[nix009-B108] Scarone S, Manzone ML, Gambini O The dream as a model for psychosis: an experimental approach using bizarreness as a cognitive marker. Schizophrenia Bull 2008;34:515–522. doi:10.1093/schbul/sbm116. 10.1093/schbul/sbm116 PMC2632423 17942480

[nix009-B109] Schmitz TW, Kawahara-Baccus TN, Johnson SC. Metacognitive evaluation, self-relevance, and the right prefrontal cortex. Neuroimage 2004;22:941–947. doi:10.1016/j.neuroimage. 2004.02.018. 15193625 10.1016/j.neuroimage.2004.02.018

[nix009-B110] Schooler JW. Re-representing consciousness: dissociations between experience and meta-consciousness. Trends Cogn Sci 2002;6:339–344. doi:10.1016/s1364-6613(02)01949-6. 12140084 10.1016/s1364-6613(02)01949-6

[nix009-B111] Schwartz S. Life goes on in dreams. Sleep 2010;33:15–6. doi:10.1093/sleep/33.1.15. 20120615 10.1093/sleep/33.1.15 PMC2802242

[nix009-B112] Schwartz S, Maquet P. Sleep imaging and the neuro-psychological assessment of dreams. Trends Cogn Sci 2002;6:23–30. doi:10.1016/s1364-6613(00)01818-0. 11849612 10.1016/s1364-6613(00)01818-0

[nix009-B113] Siclari F, Baird B, Perogamvros L The neural correlates of dreaming. Nat Neurosci 2017. doi:10.1038/nn.4545. 10.1038/nn.4545 PMC5462120 28394322

[nix009-B114] Singer JL, Antrobus JS. Eye movements during fantasies: imagining and suppressing fantasies. Arch Gen Psychiatry 1965;12:71–6. doi:10.1001/archpsyc.1965.01720310073009. 14221693 10.1001/archpsyc.1965.01720310073009

[nix009-B115] Solms M. The Neuropsychology of Dreams: A Clinico-anatomical Study. Brighton, UK: Psychology Press, 1997. doi:10.4324/9781315806440.

[nix009-B116] Solms M. Dreaming and REM sleep are controlled by different brain mechanisms. Behav Brain Sci 2000;23:843–850. doi:10.1017/s0140525x00003988. 11515144 10.1017/s0140525x00003988

[nix009-B117] Spoormaker VI, Czisch M, Dresler M. Lucid and non-lucid dreaming: thinking in networks. Int J Dream Res 2010;3:49–51. doi:10.11588/ijodr.2010.1.597.

[nix009-B118] Spoormaker VI, Van Den Bout J. Lucid dreaming treatment for nightmares: a pilot study. Psychother Psychosomat 2006;75:389–394. doi:10.1159/000095446. 10.1159/000095446 17053341

[nix009-B119] Spoormaker VI, van den Bout J, Meijer EJ. Lucid dreaming treatment for nightmares: a series of cases. Dreaming 2003;13:181–6. doi:10.1023/a:1025325529560. 10.1159/000095446 17053341

[nix009-B120] Stickgold R, Malia A, Fosse R Brain–mind states: I. Longitudinal field study of sleep/wake factors influencing mentation report length. Sleep 2001;24:171–9. doi:10.1093/sleep/24.2.171. 11247053 10.1093/sleep/24.2.171

[nix009-B121] Stickgold R, Malia A, Maguire D Replaying the game: hypnagogic images in normals and amnesics. Science 2000;290:350–353. doi:10.1126/science.290.5490.350. 11030656 10.1126/science.290.5490.350

[nix009-B122] Strauch I, Meier B. In Search of Dreams: Results of Experimental Dream Research. Albany, NY: SUNY Press, 1996.

[nix009-B123] Stumbrys T, Erlacher D. Lucid dreaming during NREM sleep: two case reports. Int J Dream Res 2012;5:151–155. doi:10.11588/ijodr.2012.2.9483.

[nix009-B124] Stumbrys T, Erlacher D, Schädlich M Induction of lucid dreams: a systematic review of evidence. Conscious Cogn 2012;21:1456–75. doi:10.1016/j.concog.2012.07.003. 22841958 10.1016/j.concog.2012.07.003

[nix009-B125] Stumbrys T, Erlacher D, Schredl M. Testing the involvement of the prefrontal cortex in lucid dreaming: a tDCS study. Conscious Cogn 2013;22:1214–22. doi:10.1016/j.concog.2013.08.005. 24021850 10.1016/j.concog.2013.08.005

[nix009-B126] Stuss DT, Picton TW, Alexander MP. Consciousness, self-awareness, and the frontal lobes In: Salloway SP, Malloy PF, Duffy JD (eds), The Frontal Lobes and Neuropsychiatric Illness. Washington, DC: American Psychiatric Publishing, 2001, 101–09.

[nix009-B127] Suzuki H, Uchiyama M, Tagaya H Dreaming during non-rapid eye movement sleep in the absence of prior rapid eye movement sleep. Sleep 2004;27:1486–90. doi:10.1093/sleep/27.8.1486. 15683138 10.1093/sleep/27.8.1486

[nix009-B128] Thompson E. Dreamless sleep, the embodied mind, and consciousness-the relevance of a classical Indian debate to cognitive science. In: Metzinger T, Windt JM (eds.), *Open MIND: 37(T)* Frankfurt am Main: MIND Group. 2015. doi:10.15502/9783958570351.

[nix009-B129] Tyson PD, Ogilvie RD, Hunt HT. Lucid, prelucid, and nonlucid dreams related to the amount of EEG alpha activity during REM sleep. Psychophysiology 1984;21:442–451. doi:10.1111/j.1469-8986.1984.tb00224.x. 6463177 10.1111/j.1469-8986.1984.tb00224.x

[nix009-B130] Underwood E. How to build a dream-reading machine. Science 2013;340:21–27. doi:10.1126/science.340.6128.21. 23559230 10.1126/science.340.6128.21

[nix009-B131] Valli K, Revonsuo A. The threat simulation theory in light of recent empirical evidence: a review. Am J Psychol, 2009, 17–38. 19353929

[nix009-B132] Valli K, Revonsuo A, Pälkäs O The threat simulation theory of the evolutionary function of dreaming: evidence from dreams of traumatized children. Conscious Cogn 2005;14:188–218. doi:10.1016/s1053-8100(03)00019-9. 15766897 10.1016/S1053-8100(03)00019-9

[nix009-B133] Vogel GW, Barrowclough B, Giesler DD. Limited discriminability of REM and sleep onset reports and its psychiatric implications. Arch Gen Psychiatry 1972;26:449–55. doi:10.1001/archpsyc.1972.01750230059012. 4336352 10.1001/archpsyc.1972.01750230059012

[nix009-B134] Voss U, Holzmann R, Hobson A Induction of self awareness in dreams through frontal low current stimulation of gamma activity. Nat Neurosci 2014;17:810–812. doi:10.1038/nn.3719. 24816141 10.1038/nn.3719

[nix009-B135] Voss U, Holzmann R, Tuin I Lucid dreaming: a state of consciousness with features of both waking and non-lucid dreaming. Sleep 2009;32:1191–200. doi:10.1093/sleep/32.9.1191. 19750924 10.1093/sleep/32.9.1191 PMC2737577

[nix009-B136] Voss U, Schermelleh-Engel K, Windt J Measuring consciousness in dreams: the lucidity and consciousness in dreams scale. Conscious Cogn 2013;22:8–21. doi:10.1016/j.concog.2012.11.001. 23220345 10.1016/j.concog.2012.11.001

[nix009-B137] Voss U, Voss GA, neurobiological model of lucid dreaming In: Hurd R, Bulkeley K (eds.), Lucid Dreaming: New Perspectives on Consciousness in Sleep. Santa Barbara, CA: Praeger Publishers, 2014, 23–36.

[nix009-B138] Wamsley EJ, Hirota Y, Tucker MA Circadian and ultradian influences on dreaming: a dual rhythm model. Brain Res Bull 2007;71:347–54. doi:10.1016/j.brainresbull.2006.09.021. 17208651 10.1016/j.brainresbull.2006.09.021

[nix009-B139] Wamsley EJ, Perry K, Djonlagic I Cognitive replay of visuomotor learning at sleep onset: temporal dynamics and relationship to task performance. Sleep 2010;33:59–68. doi:10.1093/sleep/33.1.59. 20120621 10.1093/sleep/33.1.59 PMC2802248

[nix009-B140] Wen H, Shi J, Zhang Y *Neural Encoding and Decoding with Deep Learning for Dynamic Natural Vision*, 2016 *arXiv.* arXiv: 1608.03425. 10.1093/cercor/bhx268 PMC6215471 29059288

[nix009-B141] Windt JM. Reporting dream experience: why (not) to be skeptical about dream reports. Front Hum Neurosci 2013;7. doi:10.3389/fnhum.2013.00708. 10.3389/fnhum.2013.00708 PMC3819526 24223542

[nix009-B142] Windt JM. Just in time—dreamless sleep experience as pure subjective temporality – A commentary on Evan Thompson. In: Metzinger T, Windt JM (eds.), *Open MIND: 37(C)*. Frankfurt am Main: MIND Group, 2015. doi: 10.15502/9783958571174.

[nix009-B143] Windt JM, Metzinger T. The philosophy of dreaming and self-consciousness: what happens to the experiential subject during the dream state? In: McNamara P, Barrett D (eds.), The New Science of Dreaming. Westport, CT: Praeger Publishers, 2007, 193–247.

[nix009-B144] Windt JM, Nielsen T, Thompson E. Does consciousness disappear in dreamless sleep? Trends Cogn Sci 2016;20:871–82. doi:10.1016/j.tics.2016.09.006. 27765517 10.1016/j.tics.2016.09.006

[nix009-B145] Windt JM, Noreika V. How to integrate dreaming into a general theory of consciousness—a critical review of existing positions and suggestions for future research. Conscious Cogn 2011;20:1091–107. doi:10.1016/j.concog.2010.09.010. 20933438 10.1016/j.concog.2010.09.010

[nix009-B146] Wolman RN, Kozmová M. Last night I had the strangest dream: varieties of rational thought processes in dream reports. Conscious Cogn 2007;16:838–49. doi:10.1016/j.concog.2006.09.009 17158070 10.1016/j.concog.2006.09.009

[nix009-B147] Zink N, Pietrowsky R. Theories of dreaming and lucid dreaming: an integrative review towards sleep, dreaming and consciousness. Int J Dream Res 2015;8:35–53. doi:10.11588/ijodr.2015.1.17811.

[nix009-B148] Zurawel G, Shamir I, Slovin H. Reconstruction of shape contours from V1 activity at high resolution. NeuroImage 2016;125:1005–12. doi:10.1016/j.neuroimage.2015.10.072. 26518630 10.1016/j.neuroimage.2015.10.072

